# Tumour-targeted nanomedicines: principles and practice

**DOI:** 10.1038/sj.bjc.6604483

**Published:** 2008-07-22

**Authors:** T Lammers, W E Hennink, G Storm

**Affiliations:** 1 Department of Pharmaceutics, Utrecht Institute for Pharmaceutical Sciences, Utrecht University, Sorbonnelaan 16, Utrecht 3584 CA, The Netherlands

**Keywords:** drug targeting, nanomedicines, liposomes, polymers

## Abstract

Drug targeting systems are nanometre-sized carrier materials designed for improving the biodistribution of systemically applied (chemo)therapeutics. Various different tumour-targeted nanomedicines have been evaluated over the years, and clear evidence is currently available for substantial improvement of the therapeutic index of anticancer agents. Here, we briefly summarise the most important targeting systems and strategies, and discuss recent advances and future directions in the development of tumour-targeted nanomedicines.

Over the past few decades, our knowledge on the aetiology of cancer has increased exponentially (Hanahan and Weinberg, 2000). This improved understanding of the processes that are at the heart of malignant transformation and tumorigenesis has resulted in the development of several new classes of antitumour therapeutics. In addition to classical chemotherapeutic agents (like doxorubicin, cisplatin and paclitaxel), these so-called ‘molecularly targeted therapeutics’ (like growth factor receptor inhibitors, proteasome inhibitors, histone deacetylase inhibitors and anti-angiogenic agents) have enriched the therapeutic armoury with their ability to more selectively interfere with certain ‘hallmarks of cancer’. An important, but often neglected property that such second-generation agents share with classical chemotherapeutic drugs, however, is their unfavourable biodistribution upon intravenous administration: the agents are generally rapidly cleared from the circulation, and only a very small fraction reaches the tumour site. Moreover, in certain situations, reaching the tumour is not enough: the drug may be cleared from the tumour too rapidly and may not be available long enough to display a strong therapeutic effect. Also, the physicochemical properties of the drug may make it difficult for the drug to enter the target cells. Tumour-targeted nanomedicines are drug delivery systems being developed in oncology to improve drug performance by overcoming such limitations (Table 1). Their most striking feature is their ability to target a drug to the tumour site, thereby enhancing tumoral drug levels (*site-specific delivery*; aiming for enhanced antitumour activity), and/or to direct a drug away from those body sites that are particularly sensitive to the toxic effects of the drug (*site-avoidance delivery*; aiming for reduced damage to normal tissues). In this review, we briefly address the most important nanomedicine systems and strategies, summarise the clinical status and highlight future directions.

## Passive drug targeting

**Figure 1:**
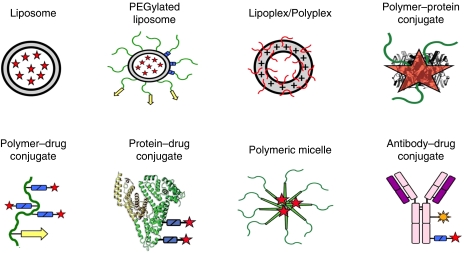
Examples of clinically used tumour-targeted nanomedicines. Representative examples of clinically used tumour-targeted nanomedicines. Liposomal bilayers are depicted in grey, polymers and polymer-coatings in green, biodegradable linkers (for releasing drugs and polymer coatings) in blue, targeting ligands in yellow, antibody fragments in purple, radionuclides in orange and the conjugated or entrapped (chemo)therapeutic agents in red.

**Figure 2:**
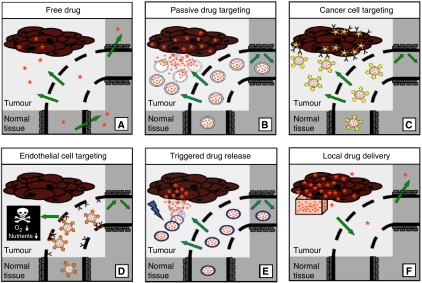
Overview of the clinically most relevant drug targeting strategies. (**A**) Upon the intravenous injection of a low-molecular-weight (chemo)therapeutic agent, which is often rapidly cleared from blood, only low levels of the drug accumulate in tumours and in tumour cells, whereas their localisation to certain healthy organs and tissues can be relatively high. (**B**) Upon the implementation of a passively targeted drug delivery system, by virtue of the enhanced permeability and retention (EPR) effect, the accumulation of the active agent in tumours and in tumour cells can be increased substantially. (**C**) Active drug targeting to internalization-prone cell surface receptors (over)expressed by cancer cells generally intends to improve the cellular uptake of the nanomedicine systems, and can be particularly useful for the intracellular delivery of macromolecular drugs, such as DNA, siRNA and proteins. (**D**) Active drug targeting to receptors (over)expressed by angiogenic endothelial cells aims to reduce blood supply to tumours, thereby depriving tumour cells from oxygen and nutrients. (**E**) Stimuli-sensitive nanomedicines, such as Thermodox, can be activated (i.e., induced to release their contents) by externally applied physical triggers, such as hyperthermia, ultrasound, magnetic fields and light. (**F**) In cases in which tumours are easily accessible, for example during surgery, sustained-release delivery devices can be implanted or injected directly into (the irresectable parts of the) tumours.

Tumour-targeted nanomedicines currently in clinical use are shown in Figure 1. Most of these systems utilise the so-called ‘passive targeting’ concept, with the exception of antibodies and their fragments, which use a receptor recognition motif for improving the delivery of the drug (through ‘active targeting’). By designing the systems such that a long circulation time is achieved, significant accumulation in tumours is obtained, especially in those tumour areas with active angiogenesis. Passive targeting refers to the substantial extravasation of the nanomedicine-associated drug into the interstitial fluid at the tumour site, exploiting the locally increased vascular permeability (Figure 2B). In addition, solid tumours tend to lack functional lymphatics, and extravasated (nano)materials are retained within the tumour site for prolonged periods of time. The exploitation of this so-called ‘enhanced permeability and retention’ (EPR) effect is currently the most important strategy for improving the delivery of low-molecular-weight (chemo)therapeutic agents to tumours (Maeda *et al*, 2000; Torchilin, 2005; Duncan, 2006).

### Liposomes

Liposomes are frontrunners among the nanomedicine systems developed so far (Bangham *et al*, 1965; Gregoriadis, 1976). Liposomes are self-assembling colloid structures composed of lipid bilayers surrounding (an) aqueous compartment(s), and can encapsulate a wide variety of (chemo)therapeutic agents. Myocet and Caelyx (Doxil in the United States) were among the first of such lipid self-assemblies to be approved by the regulatory authorities (Table 2). Both products contain doxorubicin, but differ particularly in the presence of a ‘stealth’ coating: the former refers to doxorubicin entrapped in uncoated liposomes, and the latter to liposomes surface-modified (or ‘sterically stabilized’) with poly(ethylene glycol) (PEG) to reduce rapid recognition by the reticuloendothelial system, and thereby to prolong circulation time (Drummond *et al*, 1999; Torchilin, 2005).

The pharmacokinetic benefits of liposomal drug encapsulation can be illustrated as follows: for free doxorubicin, an elimination half-life time of 0.2 h and an AUC (area under the curve) of 4 *μ*g hml^−1^ were found in patients, as compared with 2.5 h and 45 *μ*g h ml^−1^ for Myocet, and with 55 h and 900*μ*g  h ml^−1^ for Caelyx, respectively (Hofheinz *et al*, 2005). Both in animal models and in patients, such (liposome-mediated) improvements in AUC have been shown to result in significant improvements in (EPR-mediated) drug targeting to tumours (Drummond *et al*, 1999; Torchilin, 2005). However, thus far, the primary justification for approving liposomal anthracyclines has been their ability to attenuate drug-related toxicity (e.g., cardiomyopathy, bone marrow depression, alopecia and nausea), rather than to enhance antitumour efficacy. A phase III head-to-head comparison of free doxorubicin *vs* Myocet in patients with metastatic breast cancer, for instance, demonstrated in this regard that at comparable response rates (RR: 26% for both) and progression-free survival times (PFS: 4 months for both), the incidence of cardiac events (29 *vs* 13%) and of congestive heart failure (8 *vs* 2%) were significantly lower for Myocet (Harris *et al*, 2002). Also for Caelyx, significantly reduced cardiomyopathy was observed, whereas its response rates, its PFS times and its overall survival times were always at least comparable with those of the free drug (Drummond *et al*, 1999; Hofheinz *et al*, 2005; Torchilin, 2005). In certain specific cases, for example in patients suffering from AIDS-related Kaposi's sarcomas, which are characterized by a dense and highly permeable vasculature, Caelyx not only reduced the toxicity of the intervention but also substantially improved its efficacy: as compared with the formerly standard combination regimen ABV (i.e., adriamycin (doxorubicin), bleomycin and vincristine), which produced a partial response in 31 out of 125 patients (RR=25%), Caelyx achieved 1 complete response and 60 partial responses (RR=46%; Northfelt *et al*, 1998). Caelyx has consequently been approved for Kaposi's sarcoma, and is currently also marketed for metastatic breast cancer, advanced ovarian cancer and multiple myeloma.

Besides Myocet and Caelyx, several other liposomal nanomedicines have been evaluated over the years, including, for example, non-PEGylated liposomal daunorubicin (DaunoXome) and vincristine (Onco-TCS), PEGylated liposomal cisplatin (SPI-77) and lurtotecan (OSI-211), and lipoplexes, such as Allovectin and LErafAON, in which cationic lipids are used to complex, carry, protect and deliver genetic material, such as plasmid DNA and antisense oligonucleotides (Table 2). Thermodox, a temperature-sensitive version of liposomal doxorubicin (that can be triggered to release its contents; see Figure 2E), has also recently entered clinical trials. At the preclinical level, numerous additional liposomal nanomedicines have been tested, aiming not only to establish novel carrier–drug combinations (Schiffelers *et al*, 2006), but also to improve the efficacy of already existing formulations, for example, by optimising the composition of the lipid bilayer (Drummond *et al*, 1999), or by the nature or density of the polymeric stealth coatings (Romberg *et al*, 2007).

### Polymers

Ten years after the first report on liposomes (Bangham *et al*, 1965), and coinciding with the appreciation of their clinical potential (Gregoriadis, 1976), natural and synthetic polymers started to attract attention as drug delivery systems. Conceptualised by Ringsdorf (1975), it was envisioned that polymeric macromolecules can be conjugated to pharmacologically active agents by means of linkers that are stable in blood, but labile in the acidic and/or enzymatic conditions typical of, for example, the tumour microenvironment or certain intracellular compartments. These so-called ‘polymer therapeutics’ have been shown to passively accumulate in tumours by means of the EPR effect, and to be able to beneficially affect the therapeutic index of attached low-molecular-weight agents (Duncan, 2006).

In 1994, a conjugate called PK1 was the first tumour-targeted polymeric prodrug to enter clinical trials. In PK1, doxorubicin is conjugated to the prototypic polymeric drug carrier PHPMA (poly(*N*-(2-(hydroxypropyl)methacrylamide)) through an enzymatically cleavable tetrapeptide spacer (GFLG). Like Myocet and Caelyx, PK1 primarily improved the therapeutic index of doxorubicin by attenuating its (cardio)toxicity (Duncan, 2006). This is exemplified by the remarkably high maximum tolerated dose observed for PK1 in clinical trials, being >5 times higher than that determined for free doxorubicin (320 *vs* 60 mg m^−2^; Vasey *et al*, 1999). Following this proof of principle, PK1 progressed into phase II evaluation, and several additional polymer therapeutics entered clinical trials (Table 2).

In 2005, Abraxane (i.e., albumin-bound paclitaxel) was the first passively tumour-targeted polymeric nanomedicine to gain Food and Drug Administration (FDA) approval. Evidence for an advantage of Abraxane over the standard, Cremophor-formulated version of paclitaxel has been provided by a large phase III trial in which >400 women with metastatic breast cancer were randomized to receive either Abraxane (260 mg m^−2^ given as a 30-min infusion, without premedication) or the free drug (i.e., Taxol; 175 mg m^−2^ given as a 3-h infusion, with standard steroid and antihistamine premedication). As compared with Taxol, Abraxane significantly improved both the response rate (33 *vs* 19%) and the PFS time (23 *vs* 17 weeks) of systemic taxane treatment (Gradishar *et al*, 2005), and at the same time, it also attenuated its toxicity: the incidence of grade 4 neutropenias was significantly lower for Abraxane (9 *vs* 22%), despite the 50% higher dose, and no hypersensitivity reactions were observed, despite the absence of premedication (Gradishar *et al*, 2005).

Besides Abraxane and PK1, a number of additional polymeric nanomedicines have been evaluated clinically (Table 2). Oncaspar, for instance, in which the polymer PEG is conjugated to the protein L-asparaginase (to decrease allergic reactions and frequency of administration), has been used for treating patients with acute lymphoblastic leukemia for >10 years; Zinostatin, that is, PSMA-bound neocarcinostatin, has been approved in Japan for the treatment of liver cancer; and Xyotax, that is, PLGA-conjugated paclitaxel, is in phase III evaluation for ovarian and non-small-cell lung cancer. In addition to such conventional polymer–drug, polymer–protein and protein–drug conjugates, several novel types of polymeric nanomedicines have also recently entered clinical trials, including cationic polyplexes for DNA and siRNA delivery (De Smedt *et al*, 2001; Schiffelers *et al*, 2004), dendrimers (Bai *et al*, 2006), and polymeric micelles (Nishiyama and Kataoka, 2006).

## Active drug targeting

In active drug targeting, targeting ligands are attached to drugs and drug delivery systems to act as homing devices for binding to receptor structures expressed at the target site (Allen, 2002; Park *et al*, 2004). Antibody–drug conjugates targeted to, for example, CD20, CD25 and CD33, which are (over)expressed in non-Hodgkin's lymphoma, T-cell lymphoma and acute myeloid leukaemia, respectively, have been successfully used for delivering radionuclides (Zevalin), immunotoxins (Ontak) and antitumour antiobiotics (Mylotarg) more selectively to tumour cells (Table 2).

Antibodies, antibody fragments and peptides have also been used as targeting moieties for drug delivery systems. Clinical evidence in support of this strategy, however, is scarce, and has to date only been provided for galactosamine-targeted PHPMA-doxorubicin (PK2) (Seymour *et al*, 2002) and GAH-targeted doxorubicin-containing immunoliposomes (MCC-465) (Matsumura *et al*, 2004): for the former, responses were observed in 3 out of 31 patients with liver cancer (with one partial remission lasting for >47 months), and for the latter, disease stabilisation was detected in 10 out of 18 patients with gastric cancer (but no obvious reductions in tumour size).

Preclinically, a much larger number of studies have dealt with actively targeted nanomedicines, and several general principles have emerged. In most cases in which the nanosized carrier materials were targeted to receptors (over)expressed by cancer cells, for instance, the observed improvements in antitumour efficacy were found to be due to an enhanced cellular internalisation of the agents, rather than to an increased tumour accumulation (Figure 2C; Park *et al*, 2004; Kirpotin *et al*, 2006). The fact that improving cellular internalisation can – at least under certain circumstances – improve the efficacy of systemic anticancer therapy has resulted in the design of delivery systems targeted to endocytosis-prone surface receptors, such as the transferrin receptor, the folate receptor and EGFR (Allen, 2002; Torchilin, 2005; Duncan, 2006). In addition, it has stimulated research into the use of cell-penetrating peptides and protein-transduction domains, such as oligo-arginine and TAT, to enable the internalisation of agents that would otherwise not be taken up effectively by cancer cells (Gupta *et al*, 2005).

Destruction of the endothelium in solid tumours can result in the death of tumour cells induced by the lack of oxygen and nutrients. This observation, together with the high accessibility of luminal surface receptors, has led to the design of nanomedicines actively targeted to tumour endothelial cells (Figure 2D). Ligands used to target drugs and/or drug delivery systems to tumour blood vessels include the antibody fragment L19 (Neri and Bicknell, 2005), which uses the EDB domain of the oncofetal protein fibronectin to home to angiogenic vasculature, and several cyclic and linear derivatives of the oligopeptides RGD and NGR, which bind to angiogenic endothelium through the integrins α2b*β*3, αv*β*3 and α5*β*1, and through aminopeptidase-N, respectively. Recent data obtained in our group with RGD-targeted liposomes containing vascular targeting agents showed widespread central necrosis in established experimental tumours (Fens *et al*, submitted). Opposite to the short-lasting antitumour effects obtained with the free agent, endothelial cell-targeted liposomal delivery halted tumour progression for significantly prolonged periods of time. Further preclinical and clinical studies on the efficacy of tumour vasculature-targeted nanomedicines are eagerly awaited. If such nanomedicines ultimately prove in the clinic to be efficacious with manageable side effects, combination therapies together with radiation, chemotherapeutic agents and/or antiangiogenic drugs are anticipated to attack the thin film of viable tumour cells in the periphery of the tumour, which usually survives when vascular targeting agents are applied as anticancer therapeutics.

## Future directions

In this review, we have primarily restricted ourselves to tumour-targeted nanomedicines designed for the improved delivery of already established, low-molecular-weight chemotherapeutics. Many of the new drugs arising from advances in biotechnology, however, are macromolecules, such as proteins and nucleic acids. The clinical development of these challenging and often fragile molecules will likely also profit substantially from the attributes of targeted nanomedicines, providing these complex molecules, for example, with protection against degradation and elimination, and with improved access to target cells and tissues.

In the document ‘Forward Look on Nanomedicine’, the European Science Foundation included in their definition of the discipline of nanomedicine not only the use of nanometer-sized materials for the treatment but also for the diagnosis of diseases. Regarding the latter aspect, the development of high-resolution imaging techniques (such as, MRI and PET) for the rapid, noninvasive monitoring of the *in vivo* fate and performance of targeted nanomedicines is currently receiving intense attention, and will certainly facilitate the implementation of imaging-guided drug delivery to promote the optimal use of (tumour-) targeted nanomedicines.

Additional areas likely to receive considerable attention in the years to come are:

1. the design of systems that are able to respond to externally applied stimuli, such as, hyperthermia, ultrasound, light and magnetic fields, and that can be triggered to release their contents (like Thermodox; Figure 2E);
2. the targeting of agents other than conventional chemotherapeutic drugs to tumours, such as, anti-inflammatory agents (e.g., corticosteroids) to inhibit tumour-associated inflammation (Schiffelers *et al*, 2006), and siRNA to reduce the expression of proteins essential for tumour progression (Schiffelers *et al*, 2004);
3. the development of systems that are able to simultaneously deliver multiple therapeutic agents to tumours, such as temporally targeted ‘nanocells’, which first release the anti-angiogenic agent combrestatin and subsequently the chemotherapeutic agent doxorubicin (Sengupta *et al*, 2005);
4. the translation of the experience gained in oncology into applications for improving the treatment of other diseases, such as rheumatoid arthritis, Crohn's disease, autoimmune diseases and infections, which are all highly amenable to (EPR-mediated) drug targeting (Schiffelers *et al*, 2006); and
5. the establishment of treatment regimens in which tumour-targeted nanomedicines are combined with other clinically relevant treatment modalities, such as with surgery, with radiotherapy and with (standard) chemotherapy.

For obvious reasons, the latter of the above strategies has thus far received the most clinical attention. During surgery, for instance, sustained-release delivery devices, such as Gliadel (i.e., carmustine-containing polymeric wafers), can be implanted into those parts of glioblastoma lesions that cannot be removed surgically (see Figure 2F). In addition to this, also systems originally intended for systemic administration, such as polymers and liposomes, have been shown to hold potential for such local interventions (Lammers *et al*, 2006). Regarding radiotherapy, preclinical and early clinical evidence suggest that tumour-targeted nanomedicines and radiotherapy interact synergistically, with radiotherapy improving the tumour accumulation of the delivery systems, and with the delivery systems improving the interaction between radiotherapy and chemotherapy (Li *et al*, 2000; Dipetrillo *et al*, 2006; Lammers *et al*, 2008). And regarding chemotherapy, both Myocet and Caelyx have been successfully included in several different combination chemotherapy trials (Hofheinz *et al*, 2005), and also for Abraxane initial results obtained in combination regimens are promising. Combinations of molecularly targeted therapeutics with tumour-targeted therapeutics have also already been evaluated, showing, for example, that the combination of Avastin (Bevacizumab) with Abraxane produced an overall response rate of almost 50% in heavily pretreated breast cancer patients (Link *et al*, 2007).

Since the approval, in 1995, of the first tumour-targeted anticancer nanomedicine (Caelyx/Doxil, i.e., stealth liposomal doxorubicin), targeted nanomedicines have become an established addition to the anticancer drug arsenal, with several formulations presently on the market. A major limitation impeding the entry of targeted nanomedicines onto the market is that new concepts and innovative research ideas within academia are not being developed and exploited in collaboration with the pharmaceutical industry. An integrated ‘bench-to-clinic’ approach, realised within a structural collaboration between industry and academia, would strongly stimulate the progression of tumour-targeted nanomedicines towards clinical application.

## Figures and Tables

**Table 1:** Characteristics of an ideal tumour-targeted nanomedicine

(1) Increase drug localisation in the tumour through:
(a) Passive targeting
(b) Active targeting
(2) Decrease drug localisation in sensitive, non-target tissues
(3) Ensure minimal drug leakage during transit to target
(4) Protect the drug from degradation and from premature clearance
(5) Retain the drug at the target site for the desired period of time
(6) Facilitate cellular uptake and intracellular trafficking
(7) Biocompatible and biodegradable

Note that not all characteristics apply to all types of nanomedicines.

**Table 2:** Examples of clinically used tumour-targeted nanomedicines

**Compound**	**Name**	**Indication**	**Status**
Liposomal doxorubicin	Myocet, Caelyx (Doxil)	Breast, ovarian, KS	Approved
Liposomal daunorubicin	Daunoxome	Kaposi sarcoma	Approved
Liposomal vincristine	Onco-TCS	Non-hodgkin lymphoma	Approved
Liposomal cisplatin	SPI-77	Lung	Phase II
Liposomal lurtotecan	OSI-221	Ovarian	Phase II
Cationic liposomal c-Raf AON	LErafAON	Various	Phase I/II
Cationic liposomal E1A pDNA	PLD-E1A	Breast, ovarian	Phase I/II
Thermosensitive liposomal doxorubicin	ThermoDox	Breast, liver	Phase I
			
Albumin-paclitaxel	Abraxane	Breast	Approved
Albumin-methotrexate	MTX-HSA	Kidney	Phase II
Dextran-doxorubicin	DOX-OXD	Various	Phase I
PEG-L-asparaginase	Oncaspar	Leukaemia	Approved
PEG-IFN*α*2a/−IFN*α*2b	PegAsys/PegIntron	Melanoma, leukaemia	Phase I/II
PHPMA-doxorubicin	PK1	Breast, lung, colon	Phase II
Galactosamine-targeted PK1	PK2	Liver	Phase I/II
PGA-paclitaxel	Xyotax	Lung, ovarian	Phase III
			
Paclitaxel-containing polymeric micelles	Genexol-PM	Breast, lung	Phase II
Cisplatin-containing polymeric micelles	Nanoplatin	Various	Phase I
Doxorubicin-containing polymeric micelles	NK911	Various	Phase I
SN38-containing polymeric micelles	LE-SN38	Colon, colorectal	Phase I
			
^90^Yttrium-Ibritumomab tiuxetan (*α*-CD20)	Zevalin	Non-hodgkin lymphoma	Approved
DTA-IL2 fusion protein (*α*-CD25)	Ontak	T-cell lymphoma	Approved
Ozogamycin-gemtuzumab (*α*-CD33)	Mylotarg	Leukaemia	Approved
Doxorubicin-cBR96 (*α*-CD174)	SGN-15	Lung, prostate, breast	Phase II

## References

[bib1] Allen TM (2002) Ligand-targeted therapeutics in anticancer therapy. Nat Rev Cancer 2: 750–763 12360278 10.1038/nrc903

[bib2] Bai S, Thomas C, Rawat A, Ahsan F (2006) Recent progress in dendrimer-based nanocarriers. Crit Rev Ther Drug Carrier Syst 23: 437–495 17425500 10.1615/critrevtherdrugcarriersyst.v23.i6.10

[bib3] Bangham AD, Standish MM, Watkins JC (1965) Diffusion of univalent ions across the lamellae of swollen phospholipids. J Mol Biol 13: 238–252 5859039 10.1016/s0022-2836(65)80093-6

[bib4] De Smedt SC, Demeester J, Hennink WE (2001) Cationic polymer based gene delivery systems. Pharm Res 17: 113–126 10.1023/a:1007548826495 10751024

[bib5] Dipetrillo T, Milas L, Evans D, Akerman P, Ng T, Miner T, Cruff D, Chauhan B, Iannitti D, Harrington D, Safran H (2006) Paclitaxel poliglumex (PPX-Xyotax) and concurrent radiation for esophageal and gastric cancer: a phase I study. Am J Clin Oncol 29: 376–379 16891865 10.1097/01.coc.0000224494.07907.4e

[bib6] Drummond DC, Meyer O, Hong K, Kirpotin DB, Papahadjopoulos D (1999) Optimizing liposomes for delivery of chemotherapeutic agents to solid tumours. Pharmacol Rev 51: 691–743 10581328

[bib7] Duncan R (2006) Polymer conjugates as anticancer nanomedicines. Nat Rev Cancer 6: 688–701 16900224 10.1038/nrc1958

[bib8] Gradishar WJ, Tjulandin S, Davidson N, Shaw H, Desai N, Bhar P, Hawkins M, O'Shaughnessy J (2005) Phase III trial of nanoparticle albumin-bound paclitaxel compared with polyethylated castor oil-based paclitaxel in women with breast cancer. J Clin Oncol 23: 7794–7803 16172456 10.1200/JCO.2005.04.937

[bib9] Gregoriadis G (1976) The carrier potential of liposomes in biology and medicine. N Engl J Med 295: 765–770 785256 10.1056/NEJM197609302951406

[bib10] Gupta B, Levchenko TS, Torchilin VP (2005) Intracellular delivery of large molecules and small particles by cell-penetrating proteins and peptides. Adv Drug Deliv Rev 57: 637–651 15722168 10.1016/j.addr.2004.10.007

[bib11] Hanahan D, Weinberg RA (2000) The hallmarks of cancer. Cell 100: 57–70 10647931 10.1016/s0092-8674(00)81683-9

[bib12] Harris L, Batist G, Belt R, Rovira D, Navari R, Azarnia N, Welles L, Winer E (2002) Liposome-encapsulated doxorubicin compared with conventional doxorubicin in a randomized multicenter trial as first-line therapy of metastatic breast carcinoma. Cancer 94: 25–36 11815957 10.1002/cncr.10201

[bib13] Hofheinz RD, Gnad-Vogt SU, Beyer U, Hochhaus A (2005) Liposomal encapsulated anti-cancer drugs. Anticancer Drugs 16: 691–707 16027517 10.1097/01.cad.0000167902.53039.5a

[bib14] Kirpotin DB, Drummond DC, Shao Y, Shalaby MR, Hong K, Nielsen UB, Marks JD, Benz CC, Park JW (2006) Antibody targeting of long-circulating lipidic nanoparticles does not increase tumour localisation but does increase internalization in animal models. Cancer Res 66: 6732–6740 16818648 10.1158/0008-5472.CAN-05-4199

[bib15] Lammers T, Peschke P, Kühnlein R, Subr V, Ulbrich K, Huber P, Hennink W, Storm G (2006) Effect of intratumoral injection on the biodistribution and the therapeutic potential of HPMA copolymer-based drug delivery systems. Neoplasia 8: 788–795 17032495 10.1593/neo.06436 PMC1715923

[bib16] Lammers T, Subr V, Peschke P, Kühnlein R, Hennink WE, Ulbrich K, Kiessling F, Heilmann M, Debus J, Huber PE, Storm G (2008) Image-guided and passively tumor-targeted polymeric nanomedicines for radiochemotherapy. Brit J Cancer, accepted 10.1038/sj.bjc.6604561 PMC2538765 19238631

[bib17] Li C, Ke S, Wu QP, Tansey W, Hunter N, Buchmiller LM, Milas L, Charnsangavej C, Wallace S (2000) Tumor irradiation enhances the tumor-specific distribution of poly(L-glutamic acid)-conjugated paclitaxel and its antitumor efficacy. Clin Cancer Res 6: 2829–2834 10914731

[bib18] Link JS, Waisman JR, Nguyen B, Jacobs CI (2007) Bevacizumab and albumin-bound Paclitaxel treatment in metastatic breast cancer. Clin Breast Cancer 7: 779–783 18021479 10.3816/CBC.2007.n.039

[bib19] Maeda H, Wu J, Sawa T, Matsumura Y, Hori K (2000) Tumour vascular permeability and the EPR effect in macromolecular therapeutics: a review. J Control Release 65: 271–284 10699287 10.1016/s0168-3659(99)00248-5

[bib20] Matsumura Y, Gotoh M, Muro K, Yamada Y, Shirao K, Shimada Y, Okuwa M, Matsumoto S, Miyata Y, Ohkura H, Chin K, Baba S, Yamao T, Kannami A, Takamatsu Y, Ito K, Takahashi K (2004) Phase I and pharmacokinetic study of MCC-465, a doxorubicin (DXR). encapsulated in PEG immunoliposome, in patients with metastatic stomach cancer. Ann Oncol 15: 517–525 14998859 10.1093/annonc/mdh092

[bib21] Neri D, Bicknell R (2005) Tumour vascular targeting. Nat Rev Cancer 5: 436–446 15928674 10.1038/nrc1627

[bib22] Nishiyama N, Kataoka K (2006) Current state, achievements, and future prospects of polymeric micelles as nanocarriers for drug and gene delivery. Pharmacol Ther 112: 630–648 16815554 10.1016/j.pharmthera.2006.05.006

[bib23] Northfelt DW, Dezube BJ, Thommes JA, Miller BJ, Fischl MA, Friedman-Kien A, Kaplan LD, Du Mond C, Mamelok RD, Henry DH (1998) Pegylated-liposomal doxorubicin versus doxorubicin, bleomycin, and vincristine in the treatment of AIDS-related Kaposi's sarcoma: results of a randomized phase III clinical trial. J Clin Oncol 16: 2445–2451 9667262 10.1200/JCO.1998.16.7.2445

[bib24] Park JW, Benz CC, Martin FJ (2004) Future directions of liposome- and immunoliposome-based cancer therapeutics. Semin Oncol 31-S13: 96–205 10.1053/j.seminoncol.2004.08.009 15717745

[bib25] Ringsdorf H (1975) Structure and properties of pharmacologically active polymers. J Polymer Sci Polymer Symp 51: 135–153

[bib26] Romberg B, Hennink WE, Storm G (2007) Sheddable coatings for long-circulating nanoparticles. Sheddable coatings for long-circulating nanoparticles. Pharm Res 25: 55–71 17551809 10.1007/s11095-007-9348-7 PMC2190344

[bib27] Schiffelers RM, Ansari A, Xu J, Zhou Q, Tang Q, Storm G, Molema G, Lu PY, Scaria PV, Woodle MC (2004) Cancer siRNA therapy by tumour selective delivery with ligand-targeted sterically stabilized nanoparticle. Nucleic Acids Res 32: e14 15520458 10.1093/nar/gnh140 PMC528817

[bib28] Schiffelers RM, Banciu M, Metselaar JM, Storm G (2006) Therapeutic application of long-circulating liposomal glucocorticoids in auto-immune diseases and cancer. J Liposome Res 16: 185–194 16952873 10.1080/08982100600851029

[bib29] Sengupta S, Eavarone D, Capila I, Zhao G, Watson N, Kiziltepe T, Sasisekharan R (2005) Temporal targeting of tumour cells and neovasculature with a nanoscale delivery system. Nature 436: 568–572 16049491 10.1038/nature03794

[bib30] Seymour LW, Ferry DR, Anderson D, Hesslewood S, Julyan PJ, Poyner R, Doran J, Young AM, Burtles S, Kerr DJ (2002) Hepatic drug targeting: phase I evaluation of polymer-bound doxorubicin. J Clin Oncol 20: 1668–1676 11896118 10.1200/JCO.2002.20.6.1668

[bib31] Torchilin VP (2005) Recent advances with liposomes as pharmaceutical carriers. Nat Rev Drug Discov 4: 145–160 15688077 10.1038/nrd1632

[bib32] Vasey PA, Kaye SB, Morrison R, Twelves C, Wilson P, Duncan R, Thomson AH, Murray LS, Hilditch TE, Murray T (1999) Phase I clinical and pharmacokinetic study of PK1 [N-(2-hydroxypropyl)methacrylamide copolymer doxorubicin]: first member of a new class of chemotherapeutic agents-drug-polymer conjugates. Clin Cancer Res 5: 83–94 9918206

